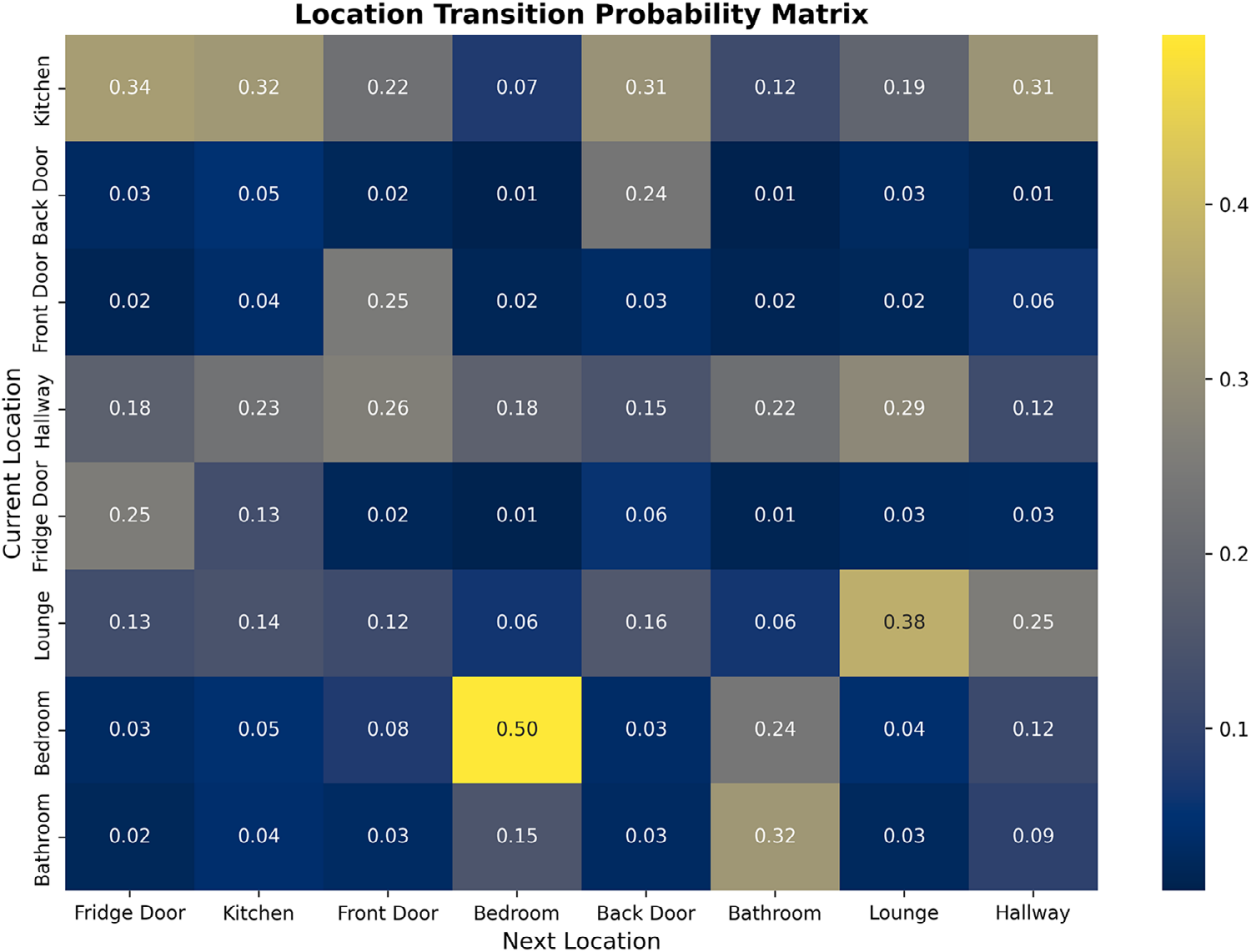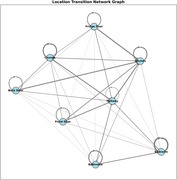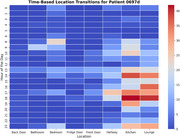# Probabilistic Framework for Detecting Irregular Movement Transitions in Alzheimer's Patients

**DOI:** 10.1002/alz70858_098515

**Published:** 2025-12-25

**Authors:** Ben George Ephrem, Abraham Varghese

**Affiliations:** ^1^ University of Technology and Applied Sciences, Muscat, Muscat, Oman; ^2^ University of Technology and Applied Sciences, Alkhuwair, Muscat, Oman

## Abstract

**Background:**

Monitoring the physical movement patterns of Alzheimer's patients is crucial due to the risk of wandering and disorientation. The currently available methods of identifying the unusual transitions and the next movement lack real‐time precision and fail to adopt patient‐specific behaviors. This study uses a probabilistic framework on the Technology Integrated Health Management (TIHM) dataset to detect movement abnormalities in Alzheimer's patients dynamically. The outcome of this study provides the caregivers with useful information and facilitates early intervention.

**Method:**

The TIHM dataset, containing spatial and temporal movement data, was used to construct patient‐specific transition sequences. A probabilistic approach using the Markov Chain model was employed to compute transition probabilities to analyze the patient's movement and was enhanced using smoothing techniques. An innovative approach of dynamic thresholding was used to identify irregular transitions with improved sensitivity. These techniques predicted the most likely subsequent movement between locations and flagged low‐probability transitions indicative of potential risks.

**Result:**

The probabilistic framework accurately predicted the next likely location for a patient, such as 'Hallway' being the most probable location from 'Bedroom'. It also detected irregular transitions like movements from 'Lounge' to 'Front Door’ and ‘Bedroom’ to ‘Fridge Door'. The transition probability matrix (Figure 1) uncovers the location‐to‐location movement trends to understand the common transitions. The location transition network graph (Figure 2) highlights critical pathways, irregular transitions and movement dynamics. The heatmap for a sample patient (Figure 3) shows the hourly activity patterns, highlighting the peak movement periods and potential anomalies. These visualizations offer a wide analysis of patient movement behavior. This provides the caretakers with precise and practical information to identify and effectively manage anomalies.

**Conclusion:**

This framework addresses the main drawbacks of conventional techniques by introducing a dynamic, patient‐specific approach to tracking the mobility patterns in Alzheimer's patients. It supports real‐time actions to increase safety and lower risks by improving carer decision‐making through its predictive capabilities. If integrated with smart healthcare systems, this framework could revolutionize Alzheimer's patient care by fostering a safer and more responsive care‐giving environment.